# Torsion of the testis: a new risk factor for testicular cancer.

**DOI:** 10.1038/bjc.1987.21

**Published:** 1987-01

**Authors:** C. E. Chilvers, M. C. Pike, M. J. Peckham


Br. J. Cancer (1987), 55, 105–106 © The Macmillan Press Ltd., 1987

SHORT COMMUNICATION

### Torsion of the testis: A new risk factor for testicular cancer

C.E.D. Chilvers[1], M.C. Pike[2] & M.J. Peckham[3]

[1]*Section of Epidemiology, Institute of Cancer Research, Sutton, Surrey SM2 5PX;* [2]*Imperial Cancer Research Fund Epidemiology Unit, Radcliffe Infirmary, Oxford OX2 6HE and* [3]*Academic Radiotherapy Unit, Institute of Cancer Research, Sutton, Surrey SM2 5PX, UK.*

Using data from the Hospital In-patient Enquiry (HIPE) Coggon and Nelms (1984) reported a substantial increase in operations for torsion of the testis between 1968 and 1980. The cumulative discharge rate (for ages 0 to 34) increased 2.3-fold from 0.35% in 1968 to 0.82% in 1980. A subsequent investigation of cases in the Wessex Health Region by these authors confirmed this increase and could find no artefactal explanation for it (Nelms & Coggon, 1986). Undescended testis has also been increasing in incidence over this period (Chilvers *et al.*, 1984; John Radcliffe Hospital Cryptorchidism Study Group, 1986) and, since torsion has been reported as being associated with an anatomical abnormality (Scorer & Farrington, 1971), a common aetiological factor may be responsible for the increase in both conditions (Coggon & Nelms, 1984).

Undescended testis is the major known risk factor for testicular cancer not only in the undescended testis itself (whether or not brought down by orchidopexy) but also for the contralateral scrotal testis (Henderson *et al.*, 1983; Pike *et al.*, 1986). This suggests that it is not the position of the testis that is important but that some underlying factor predisposes to both conditions. If there is a common aetiological factor in cryptorchidism and torsion, it is reasonable to suggest that patients with a history of torsion of the testis might also be at increased risk of developing cancer of the testis. We have investigated this possibility using data from the Royal Marsden Hospital (RMH).

The records of all 871 patients who attended the RMH for the first time with a diagnosis of testicular cancer between January 1, 1975, and December 31, 1984, were reviewed. Seven hundred and twenty four of these patients were born in the UK and were resident in the UK at the time of diagnosis; it is only for these patients that the expected number of testicular torsions can be calculated for comparison purposes using HIPE data. Almost all patients had had their orchidopexy elsewhere and were referred soon afterwards for detailed evaluation and, if necessary, further treatment. Patients with bilateral tumours were included only if they attended the RMH with their first tumour within this period. Only patients with germ-cell tumours were eligible; patients with lymphomas and sarcomas were excluded, as were patients whose tumour was not positively identified as being a testicular primary.

The case-notes of the patients were abstracted by one of two research nurses onto a standard form. The information abstracted included details of the histology of the tumour, history of undescended testis, torsion and other testicular problems. Tumours were classified as seminomas (pure seminoma with no other elements present) or teratomas (all others).

We calculated age-specific rates for operations for testicular torsion for each calendar year from 1968 to 1980 using the HIPE data (see Figure 1). Assuming that the 1968 rates applied to all previous years and the 1980 rates to all

Correspondence: C.E.D. Chilvers.
Received 4 August 1986.

**Figure 1** Discharge rate for torsion by age-group, 1968–80.

subsequent years, we calculated the number of testicular torsions that would be expected to have occurred in the 724 RMH UK patients prior to diagnosis with a testicular tumour.

Nine of the 724 UK patients had a history of testicular torsion recorded in their case-notes; one of the nine had a history of maldescent in the contralateral (malignant) testis. The expected number of cases with torsion was calculated to be 2.73: this gives a relative risk of 3.3 (exact test, 1-sided $P=0.002$), that is, we estimate that men with a history of torsion have an approximately three-fold increased risk of developing a testicular tumour. If we exclude from the 724 total patients the 69 patients with a history of cryptorchidism the observed number of cases with torsion was 8 and the expected number was 2.47; this gives a relative risk of 3.2 (exact test, 1-sided $P=0.004$).

Our assumption that the torsion rates remained steady after 1980 rather than continuing to increase makes a negligible difference to the calculated expected number of 2.73. If, as seems likely, the torsion rates before 1968 were lower than observed in 1968, then the expected number would be less than 2.73 and the relative risk of testicular cancer associated with torsion would be greater than 3.3.

The histology and ages at diagnosis of testicular cancer and torsion are given in Table I. Only one (12.5%) of the eight patients with a history of torsion and known histology had a seminoma compared to 31% (221/704 unilateral tumours) of the patients with no history of torsion; this difference is not statistically significant (exact test, 1-sided $P=0.23$). The age at diagnosis of the teratomas with a history of torsion was slightly younger (mean 25.7 years)

**Table I** Age at diagnosis of testicular torsion and subsequent germ-cell tumour

| | *Age at diagnosis of:* | | *Side of:* | |
|---|---|---|---|---|
| *Histology* | *Torsion* | *Tumour* | *Torsion* | *Tumour* |
| Teratoma | 22 | 22 | R | R |
| Teratoma | 25 | 25 | L | L |
| Teratoma | 15 | 25 | L | L |
| NK | 13 | 25 | R | L |
| Teratoma | 12 | 25 | L | R |
| Teratoma | 17 | 26 | R | R |
| Teratoma | 'child' | 28 | R | L |
| Seminoma | 22 | 28 | L | R |
| Teratoma | 18 | 29 | Both | R |

than for all teratomas (29.3 years); this difference might be at least partially accounted for by the fact that in two cases the investigation of torsion led directly to the diagnosis of the tumour.

Only three torsions were diagnosed under age 15 (see Table I), whereas it would be expected that most would be diagnosed before that age (see Figure 1). In four cases the torsion occurred in the contralateral testis: one of these four patients had an orchidectomy for torsion and a history of maldescent in his other (malignant) testis, and one other patient had an orchidectomy for torsion. The fact that torsion may occur in the contralateral testis and the discovery of the testicular tumour at the time of the torsion in two cases emphasises that the torsion itself is unlikely to have any role in the aetiology of the tumour.

The demonstration that torsion of the testis is associated with at least a 3-fold increase in the risk of testicular cancer, when taken together with the marked secular trend in torsion rates, provides further strong evidence that testicular cancer, which is already the commonest neoplasm in men aged 25–34 in England and Wales, is going to continue to increase and that urgent attempts should be made to discover the underlying cause of these disorders.

We thank Eileen Williams and Elizabeth Hilton for abstracting case-notes, Carol Hermon for computing assistance, and Doreen Folkes and Sarah Jones for preparing the manuscript. A photocopy of the number of discharges from the Hospital In-patient Enquiry was kindly supplied by Dr David Coggon. The Institute of Cancer Research receives support from the Cancer Research Campaign and the Medical Research Council.

### References

CHILVERS, C., PIKE, M.C., FORMAN, D., FOGELMAN, K. & WADSWORTH, M.E.J. (1984). Apparent doubling of frequency of undescended testis in England and Wales in 1962–81. *Lancet*, **ii**, 330.

COGGON, D. & NELMS, M. (1984). Incidence of testicular abnormalities. *Lancet*, **ii**, 747.

HENDERSON, B.E., ROSS, R.K., PIKE, M.C. & DEPUE, R.H. (1983). Epidemiology of testis cancer. In *Urologic Cancer*, Skinner, D.G. (ed) p. 237. Grune & Stratton: New York.

JOHN RADCLIFFE HOSPITAL CRYPTORCHIDISM STUDY GROUP (1986). Increase in cryptorchidism since 1960. *Br. Med. J.* (in press).

NELMS, M. & COGGON, D. (1986). Increase in hospital admissions for torsion of testis. *J. Epidemiol. Comm. Hlth.*, **40**, 76.

PIKE, M.C., CHILVERS, C. & PECKHAM, M.J. (1986). Effect of age at orchidopexy on risk of testicular cancer. *Lancet*, **i**, 1246.

SCORER, C.G. & FARRINGTON, G.H. (1971). *Congenital Deformities of the Testis and Epididymis*. Butterworth: London.


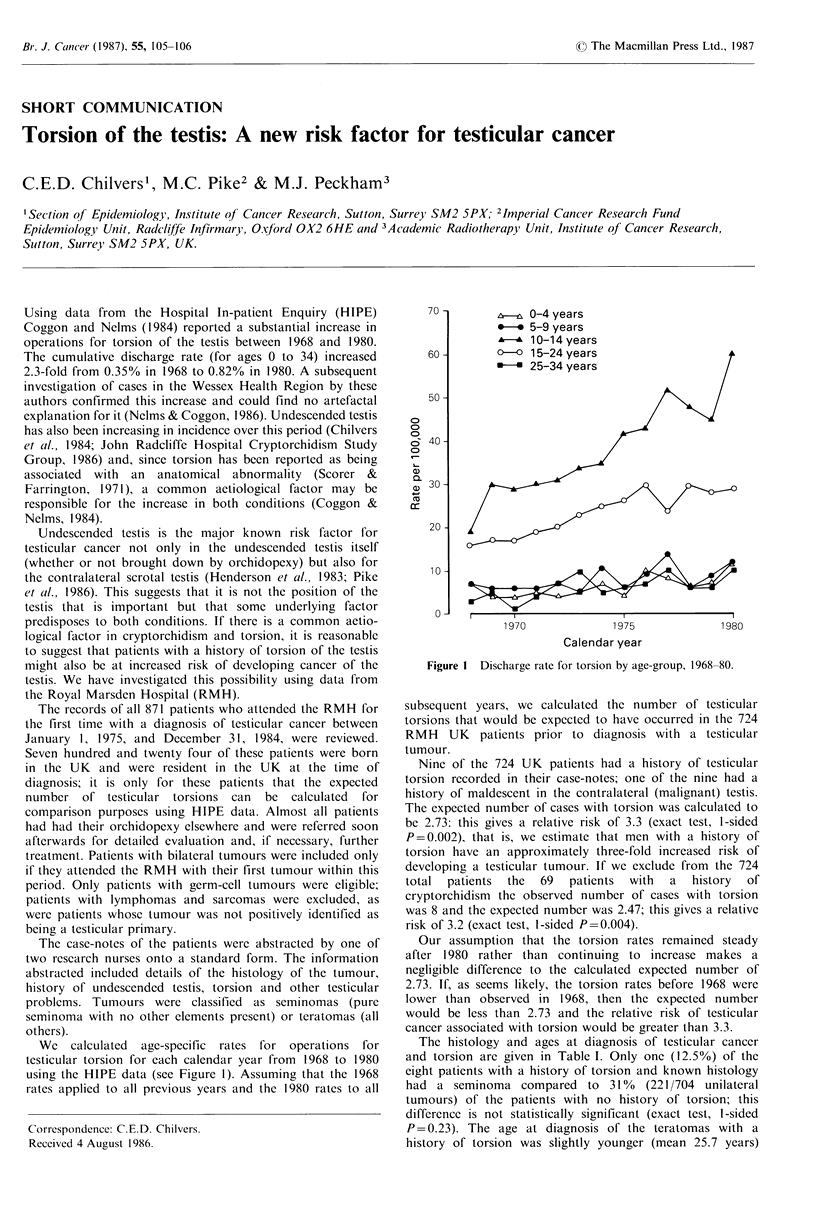

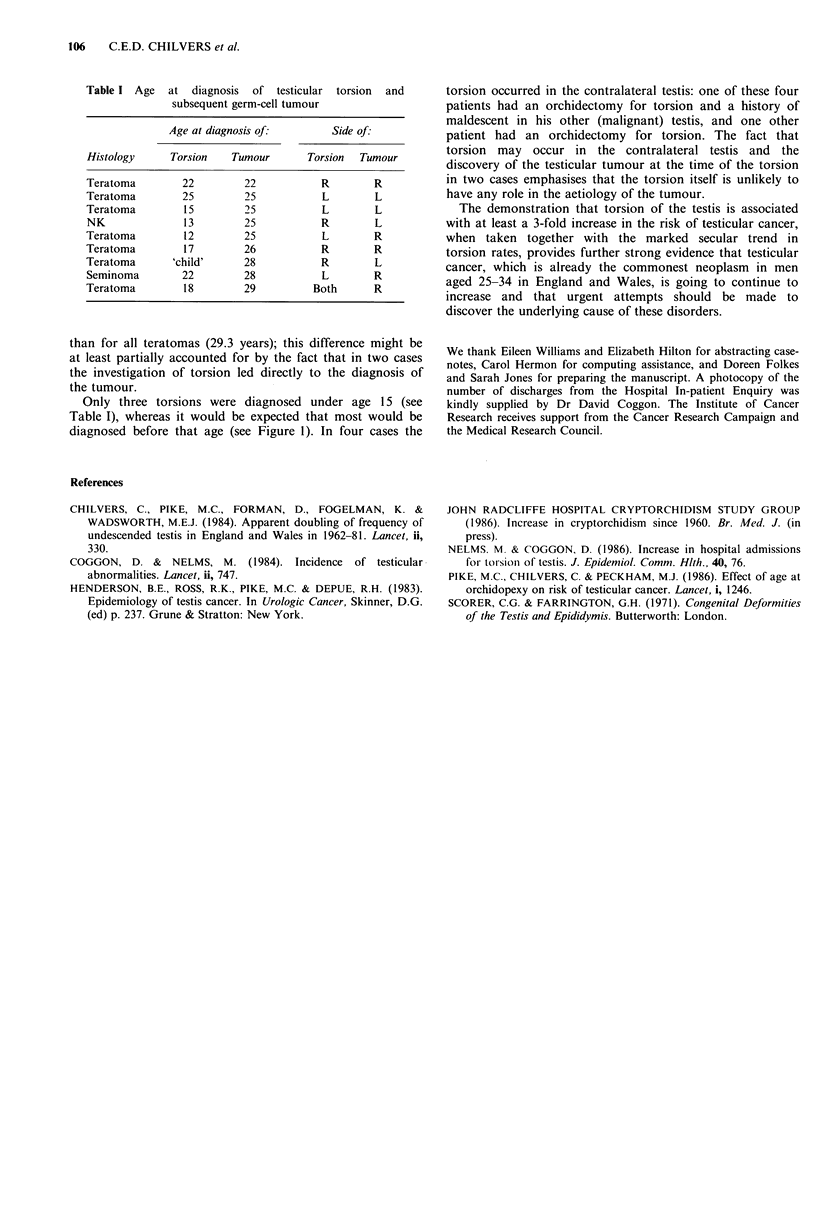


## References

[OCR_00221] Chilvers C., Pike M. C., Forman D., Fogelman K., Wadsworth M. E. (1984). Apparent doubling of frequency of undescended testis in England and Wales in 1962-81.. Lancet.

[OCR_00227] Coggon D., Nelms M. (1984). Incidence of testicular abnormalities.. Lancet.

[OCR_00241] Nelms M., Coggon D. (1986). Increase in hospital admissions for torsion of testis.. J Epidemiol Community Health.

[OCR_00245] Pike M. C., Chilvers C., Peckham M. J. (1986). Effect of age at orchidopexy on risk of testicular cancer.. Lancet.

